# Inflammatory and Anti-Inflammatory Equilibrium, Proliferative and Antiproliferative Balance: The Role of Cytokines in Multiple Myeloma

**DOI:** 10.1155/2017/1852517

**Published:** 2017-09-26

**Authors:** Caterina Musolino, Alessandro Allegra, Vanessa Innao, Andrea Gaetano Allegra, Giovanni Pioggia, Sebastiano Gangemi

**Affiliations:** ^1^Division of Hematology, Department Patologia Umana dell'Adulto e dell'Età Evolutiva, University of Messina, Località Gazzi, Via Consolare Valeria, Messina, Italy; ^2^Institute of Applied Sciences and Intelligent Systems “Eduardo Caianiello” (ISASI)-National Research Council of Italy (CNR), Messina Unit, Via Torre Bianca, Messina, Italy; ^3^School and Division of Allergy and Clinical Immunology, Department of Clinical and Experimental Medicine, University Hospital “G. Martino”, Località Gazzi, Via Consolare Valeria, Messina, Italy

## Abstract

Multiple myeloma (MM) is typically exemplified by a desynchronized cytokine system with increased levels of inflammatory cytokines. We focused on the contrast between inflammatory and anti-inflammatory systems by assessing the role of cytokines and their influence on MM. The aim of this review is to summarize the available information to date concerning this equilibrium to provide an overview of the research exploring the roles of serum cytokines in MM. However, the association between MM and inflammatory cytokines appears to be inadequate, and other functions, such as pro-proliferative or antiproliferative effects, can assume the role of cytokines in the genesis and progression of MM. It is possible that inflammation, when guided by cancer-specific Th1 cells, may inhibit tumour onset and progression. In a Th1 microenvironment, proinflammatory cytokines (e.g., IL-6 and IL-1) may contribute to tumour eradication by attracting leucocytes from the circulation and by increasing CD4 ^+^ T cell activity. Hence, caution should be used when considering therapies that target factors with pro- or anti-inflammatory activity. Drugs that may reduce the tumour-suppressive Th1-driven inflammatory immune response should be avoided. A better understanding of the relationship between inflammation and myeloma will ensure more effective therapeutic interventions.

## 1. Introduction

Multiple myeloma (MM) is a clonal B cell neoplasia that results from the growth of malignant plasma cells within the bone marrow (BM), in close connection with other cells in the bone environment. Stromal cells sustain MM cell persistence and growth [1]. Amongst them, inflammatory cells have a crucial role in tumour growth and MM progression [2].

In fact, the relationships of myeloma cells with BM stromal cells are relevant for their increased proliferation, homing pattern, and survival [2]. The BM environment and myeloma cells stimulate paracrine or autocrine secretion of several mediators. In fact, the BM microenvironment in MM subjects displays high levels of HGF, interleukin- (IL-) 2R, IL-16, EGF, and cytokines induced by interferon-*γ* (IFN-*γ*) [3]. Many of these cytokines are considered to be promoters of MM development [4–9], sometimes operating as growth factors for MM cells and sometimes promoting cellular adhesion. Other cytokines appear to increase angiogenesis or osteoclastogenesis [10–16].

It is well known that cytokines are implicated both in inflammatory and anti-inflammatory processes and are the manifestation of a system that includes genes and polymorphisms. Many of these factors that are altered in the serum or bone marrow of MM subjects have proinflammatory activity, such as IL-1, IL-6, IL-12, IL-15, IL-16, IL-17, IL-18, IL-22, IL-23, TNF-*α*, and IFN-*γ*, while others exert anti-inflammatory effects, such as IL-1R*α*, IL-4, IL-10, IL-11, TGF-*β*1, heat-shock proteins (HSPs), and lipoxin A4.

Although essential for defence against microbial infection, the inflammatory response requires stringent control because incorrect inflammatory signals and disproportionate cell death are the cause of several diseases.

Tumour growth is also associated with significant inflammation; an increase in proinflammatory cytokine levels can support the progression of neoplasia [17]. Cytokines secreted by plasma cells and T lymphocyte subsets can create an environment in the BM that fosters malignant cell development.

## 2. T Lymphocyte Subsets in Multiple Myeloma

The imbalance of T lymphocyte subsets may perform relevant roles in MM [18, 19].

Depending on the substances delivered and functions, CD4+ T cells can be grouped into several subsets comprising T helper 1 (Th1), Th2, Th17, and CD4+ CD25+ T regulatory (Treg) cells [20, 21]. Th1 cells produce interferon gamma (IFN-*γ*) and stimulate the cell-mediated immune response, while Th2 cells deliver IL-4 and inhibit the Th1 cell-mediated response. Th17 cells generate IL-17A, IL-6, and TNF-*α* implicated in stimulating inflammation [22, 23]. Treg cells repress effector T cell growth by producing TGF-*β* and IL-10, which exert immunomodulatory actions. The imbalance between Treg and Th17 cells has become a key function in inflammatory diseases.

Recently, Th17 cells have been implicated in the occurrence of MM and its complications [24–28].

The CD4+ Th1 and CD4+ Th17 subsets in subjects with MM were considerably higher than those in healthy subjects, as were the levels of T-bet and RORgamma mRNA [29].

Wang et al. noted that the numbers of another T cell type, Th22 cells, were significantly higher in peripheral blood (PB) and bone marrow (BM) of MM subjects and recovered in subjects with complete remission after treatment. Furthermore, the numbers of Th22 and Th17 cells were greater in stage III than in stages I and II MM [30].

Treg cells have a relevant function in the protection of self-tolerance and of immune responses against tumour cells. The anomalous Treg activity in MM subjects could, on the other hand, participate in the MM-related immune dysfunction [31].

The action of Tregs in the biology of MM has been studied by several authors. Nevertheless, many *in vitro* or *in vivo* data remain ambiguous. For instance, one study calculated the number of Tregs in the peripheral blood (PB) of controls versus subjects with MGUS and MM and displayed a significant decrease in the number of Treg cells. These cells were reported as dysfunctional and incapable of suppressing the growth of T lymphocytes. However, another study evaluated the number and function of Tregs in the PB and BM of controls and MM subjects and did not show a modification in the proportion of Treg cells between the two sites, between either group of subjects [32].

Huang et al. investigated the action of Tregs in the onset of MM-related kidney impairment (KI). The Tregs significantly decreased in the MM-related KI subjects compared with the controls. The number of Tregs was negatively correlated with blood urea nitrogen, serum IL-6, IL-4, *β*2-microglobulin, monoclonal immunoglobulin, BM plasma cell percentages, and proteinuria, but they were positively correlated with the glomerular filtration rate. Tregs gradually diminished as the stage of disease increased [33].

## 3. Inflammatory and Anti-Inflammatory Balance in MM

**Figure 1:**
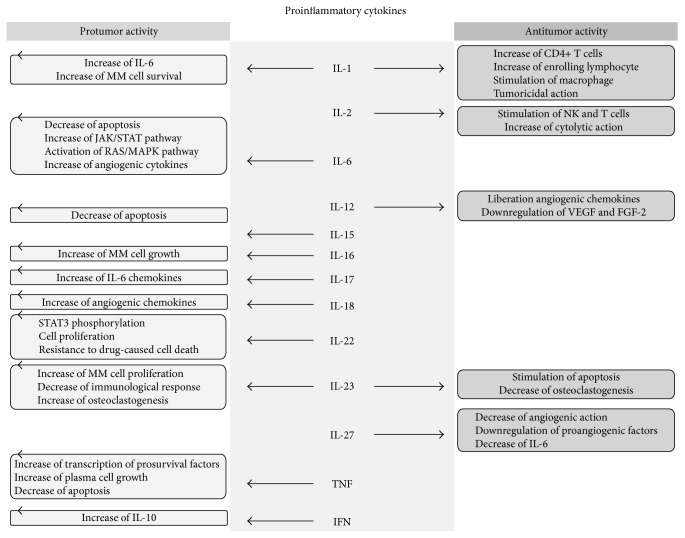
Effects of proinflammatory cytokines action on multiple myeloma cells and, therefore, on the tumour itself. Some of them have only protumour action, while for others there is a simultaneous dual mechanism of action pro and antitumour.

**Figure 2:**
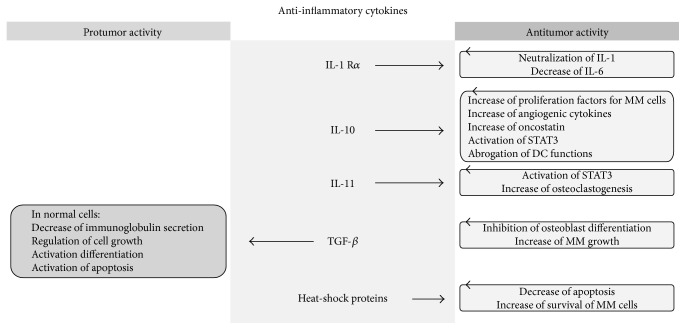
All anti-inflammatory cytokines have an antitumour effect, except for TGF-*β*.

In this review, we have concentrated our attention on the equilibrium between the inflammatory and anti-inflammatory systems by assessing the action of cytokines and their impact on MM. The aim of our work is to summarize what is known to date about this balance, providing an overview of the research examining the role of serum cytokines in MM (Figures 1 and 2).

However, as we shall demonstrate later, the MM-inflammatory cytokine increase appears to be inadequate, and other functions such as pro-proliferative or antiproliferative effects can assume the activities of cytokines in the genesis and progression of MM.

## 4. Proinflammatory Cytokines

### 4.1. IL-1

IL-1 is a powerful proinflammatory cytokine that operates as an endogenous pyrogen. It is delivered by fibroblasts, monocytes, tissue macrophages, and dendritic cells (DCs), but it is also produced by B lymphocytes, epithelial cells, and natural killer (NK) cells.

IL-1 was first cloned in the 1980s and quickly found to be a key agent in the control of inflammatory processes. The name IL-1 encompasses two cytokines, IL-1*α* and IL-1*β*, which are produced by two diverse genes. The actions of IL-1 are tightly regulated by numerous inhibitors, such as IL-1 receptor antagonist (IL-1Ra), IL-1 receptor type II (IL-1RII), and additional soluble receptors [34].

The cytokine has various potentiating effects on cell growth, differentiation, and the function of several immunocompetent cells. It plays a role in numerous inflammatory diseases by initiating immune and inflammatory responses [35].

Numerous researchers have evaluated the role of IL-1 in the pathogenesis of MM. Like mature B lymphocytes, the myeloma plasma cell generates IL-1. In the BM environment, stromal cells react to low levels IL-1 and produce large quantities of IL-6, which in turn stimulate the survival of the myeloma cells [36]. Blockade of IL-1 results in a decrease in IL-6 activity [37].

An *in vivo* work confirmed that IL-1*β* has a relevant role in the conversion of latent myeloma to active MM. The aim of this study was to decelerate or prevent progression of the disease. Subjects with latent/indolent MM at high risk of progression were treated with anakinra, an inhibitor of IL-1, for 6 months. During the treatment, there was a reduction in C-reactive protein (CRP) and a decrease in the plasma cell-labelling index. After 6 months of treatment, a low dose of dexamethasone was added. Of the 47 subjects who received anakinra, progression-free disease (PFD) was achieved after 3 years and 4 years in 8 subjects. Subjects with a reduction in serum CRP of 15% after 6 months of therapy achieved PFD after 3 years compared with 6 months in subjects with less than a 15% reduction [38].

A different inhibitor of IL-1 is the engineered P2D7KK antibody. This substance has a strong affinity for IL-1*β*, resulting in robust neutralization of human IL-1*β*. In an experimental model, P2D7KK therapy reduced MM-induced lethality; 70% of P2D7KK-treated animals survived compared with 20% in the control group. Remarkably, the survival percentage inversely correlated with the serum concentrations of IL-6, further supporting the relevant role of IL-1*β* in the pathway leading to MM [39].

### 4.2. IL-2

IL-2 is principally generated by CD8^+^ and CD4^+^ T cells. Target cells of IL-2 comprise CD4 CD8 T cells, B cells, and NK cells. IL-2 has a relevant role in T cell-dependent responses.

IL-2 was one of the first cytokines to be accepted for the treatment of tumours, despite its having one of the most complicated and, in some circumstances, incongruous roles in immune stimulation. Not only does IL-2 strongly stimulate NK and T cell growth and augment their cytolytic action, but it also sensitizes T cells to activation-induced cell death and is required for Treg cells to reduce persistent immune responses [40].

Benson et al. have established that NK cells derived from MM subjects exhibit the inhibitory receptor PD-1, whereas NK cells from healthy subjects do not show this receptor unless activated by IL-2 [41].

Emerging evidence indicates that NK cells also have anti-MM activity [42], and *in vitro* studies have revealed that allogeneic and auto NK cells have the capability to kill CD138-purified MM cells [43].

In human subjects, NK cells are controlled by killer immunoglobulin-like receptors (KIRs) that identify allotypic determinants exhibited by diverse human leucocyte antigen (*HLA*) class I alleles.

A study has revealed that administration of IL-2-activated haploidentical killer immunoglobulin-like receptor (KIR) ligand-mismatched NK cells to MM subjects was effective, and 50% of the subjects achieved nearly complete remission [44].

Moreover, it is well known that hypoxia decreases NK cell eradication of MM cell lines in an oxygen-dependent mode. During hypoxia, NK cells had a conserved capability to degranulate in response to target cells, although the rate of degranulating NK cells was slightly diminished. Preactivation of NK cells by IL-2 abolished the detrimental actions of hypoxia and augmented NKG2D expression, highlighting that NK cell-activated IL-2 can have anti-MM actions, even under hypoxic conditions [45].

### 4.3. IL-6

IL-6 is a pleiotropic cytokine implicated in the control of acute responses, immune reactions, haematopoiesis, and inflammation. It has context-dependent pro- and anti-inflammatory activities with diverse signalling pathways. IL-6 is generated by monocytes, endothelial cells, macrophages, and fibroblasts in response to diverse stimuli (TNF-*α*, IL-1, and IL-17) during systemic inflammation [46]. It stimulates the growth and activation of T cells, the differentiation of B cells, and the control of the acute-phase response [47].

IL-6 expression is generally low, and serum concentrations are normally nondetectable in the absence of inflammation.

It is implicated in lymphoid tumours and functions as a crucial growth factor for MM by decreasing apoptosis caused by growth factor withdrawal and by establishing the expression of the cell death receptor Das [48].

IL-6 binds to IL-6 receptor (IL-6R) to initiate IL-6 signalling. IL-6R, which is generated by MM cells, consists of an alpha (gp80) and a signal transducer beta (gp130) chain. This structure binds to the signal transducer membrane protein gp130, which stimulates Janus kinases/signal transducer-activator of transcription (JAKs/STAT) and the GTPase/mitogen-activated protein kinase (RAS/MAPKs) pathway. It is well known that JAK/STAT has a role in MM growth and inhibition of apoptosis, while RAS/MAPK is active in proliferation.

There is also a soluble form of gp80 (sIL-6R) that is produced either by proteolytic cleavage or by transcription of a splice variant. sIL-6R is increased in MM, with serum concentrations that are interrelated to both disease and angiogenic activity factors [49]. In fact, it has been established that the mean levels of sIL-6R, and the value of Ki-67, were considerably higher in MM compared with health subjects. Moreover, there is a positive correlation between plasma cell proliferation and diverse angiogenic cytokines, such as IL-18 and HGF, with sIL-6R [50].

Nevertheless, IL-6 not only participates in the proliferation of myeloma cells, but also is a main morbidity factor in MM subjects. Augmented IL-6 concentrations are associated with disease-related renal involvement, thrombocytosis, anaemia, bone reabsorption, and a prothrombotic state. In numerous malignancies, encompassing MM, lymphoma, and lung cancer, high serum concentrations of IL-6 have been associated with a poor prognosis [51, 52].

The cellular source of IL-6 in MM patients has long been unclear. IL-6 was first identified as an autocrine factor generated by malignant plasma cells. More recent research has shown that IL-6 is a paracrine factor delivered by the microenvironment, principally by cells from the myeloid compartment. Matthes et al. have validated that IL-6 derives from myeloid precursors. They have also confirmed that IL-6 augments the pool of myeloid cells to generate a second factor for MM cells, a proliferation-inducing ligand (APRIL). These data establish a novel motivation for IL-6 inhibition in MM subjects [53].

Because IL-6 is known as a proliferative factor in MM cells, inhibition of IL-6 signalling was established in 1991 as a therapeutic method for MM subjects. Unfortunately, the first clinical trial did not demonstrate a clear advantage; however, despite this failure, the chances of IL-6 antagonism remain still high. Siltuximab is a chimeric anti-IL-6 antibody, and it was studied for its advantageous anti-IL-6 effects on tumours, such as MM [54, 55]. Nevertheless, therapy with siltuximab intensified the possibility of upper respiratory tract infections or other adverse effects including fatigue, nausea, rash, pruritus, dyspnoea, increased weight gain, thrombocytopenia, and neutropenia [56].

Another method to reduce IL-6 production could consist of the administration of clarithromycin (CAM) (or 6-O-methyl erythromycin), a macrolide antibiotic [57].

There is evidence that CAM is a powerful inhibitor of autophagy in MM [58]. The proteasome inhibitor bortezomib (Bor) also causes autophagy. The combination of Bor and CAM caused augmented cytotoxicity compared with Bor alone [59]. Durie et al. registered a greater than 50% response rate [60]. Other authors utilizing CAM as monotherapy could not validate its activity in MM [61–64].

Since CAM reduces IL-6 secretion, it might have a direct anticancer action in MM [65, 66].

### 4.4. IL-12

IL-12 is a cytokine that is able to stimulate both innate and adaptive immunity. It causes Th1-cell cytotoxicity and has been shown to have strong immunomodulatory and antitumour activities [35].

The inflammatory activity of IL-12 has been confirmed in subjects with psoriatic arthritis (PsA). Ustekinumab is a monoclonal antibody that binds to the p40-subunit of IL-12, and ustekinumab therapy resulted in considerably improved PsA activity [67].

Nevertheless, although IL-12 is an inflammatory cytokine, in this case the prevalent activity of the cytokine seems to be protection against neoplastic disease.

IL-12 exerts its antitumour activity via immunostimulatory and antiangiogenic actions related to the production of IFN-*γ*, which stimulates the liberation of the antiangiogenic chemokines CXCL9, CXCL10, and CXCL11. Moreover, IL-12 downregulates the liberation of vascular endothelial growth factor (VEGF) and fibroblast growth factor-2 (FGF-2) [68–75].

The IL-12RB2 gene encoding the IL-12R chain acts as a tumour suppressor in neoplastic B cells from several chronic lymphoproliferative diseases and acute lymphoblastic leukaemia. Airoldi et al. have also showed that *IL-12rb2*-deficient mice develop multiorgan lymphoid infiltrates, CD138^+^ cell hyperplasia, and display IL-6 upregulation. Moreover, *IL-12rb2* knockout animals have localized lymph node plasmacytoma, which is probably related to IL-6 overexpression [76–79].

In another study, Airoldi et al. examined the function of IL-12R*β*2 in MM pathogenesis. They demonstrated that IL-12R*β*2 was present in primary MM cells but was downregulated in comparison to polyclonal plasmablastic cells and plasma cells. IL-6 reduced IL-12R*β*2 expression on plasmablastic and MM cells. Furthermore, IL-12 decreased the proangiogenic action of primary MM cells *in vitro* and reduced the tumourigenicity of the NCI-H929 cell line in mice by reducing cell growth and angiogenesis. This phenomenon depended on reduced expression of several proangiogenic genes and upregulated expression of several antiangiogenic genes, such as platelet factor-4, IFN-*γ*, IFN-*α*, and TIMP-2. Reduction of the angiogenic action of MM cells was correlated to downregulated expression of the proangiogenic genes CD13, AKT, CCL11, and vascular endothelial-cadherin, and the upregulation of an IFN-*γ*-correlated antiangiogenic pathway. Therefore, IL-12R*β*2 limits MM cell proliferation, and targeting of IL-12 to MM cells could be a novel therapeutic approach [80].

Wang et al. examined the possibility of using proteasome inhibition by Bor and immune treatment with IL-12 to augment the antitumour efficacy relative to the actions of either of those agents alone. IL-12 therapy alone caused a mild reduction in tumour volume compared with the control. Bor alone inhibited tumour proliferation, resulting in a decrease in tumour volume by ~65% after 15 days of treatment. The combination treatment produced ~75% decreases in tumour growth, considerably exceeding the decrease caused by Bor monotherapy. Tumour growth resumed following the conclusion of Bor therapy at 2 weeks, although the tumour size was still smaller than that in the NS and IL-12 animals. This rebound of tumour enlargement was entirely avoided with the combination treatment, and the tumour volume continued to decline over the time course. Moreover, combination treatment reestablished the Bor-induced functional deficiency of the NK cells relative to Bor administration alone [81].

To establish the effectiveness and toxicity of IL-12, the Eastern Oncology Cooperative Group (ECOG) conducted a phase II trial of this substance in treated subjects with plateau phase MM. Half the subjects received IL-12 in combination with vaccines against *Streptococcus pneumoniae* and *Haemophilus influenza*, and half received IL-12 at 30 days after receiving their vaccines. Of 48 subjects, there were 4 CR (8.3%). The progression-free survival and median survival were 11.4 and 42.8 months, respectively. Grade 3 or 4 nonhaematologic toxicity (63% with intravenous IL-12 and 31% with subcutaneous IL-12) was detected [82].

Finally, IL-12 alone or with GM-CSF has been utilized to increase anti-idiotype vaccines in subjects with MM [83].

### 4.5. IL-15

IL-15 is a cytokine that is able to maintain the cellular immune response, stimulating the growth of human memory T cells. IL-15 is comparable to IL-2 in terms of biological actions [84].

IL-15 binds to IL-15 receptor *α* (IL-15R*α*) and is presented in trans to the IL-2/IL-15 receptor *β* common *γ* chain (IL-15R*βγ*_c_) complex. IL-15 and IL-2 exhibit the same binding to IL-15R*βγ*_c_ and act via the STAT3 and STAT5 pathways. Nevertheless, unlike IL-2, IL-15 does not sustain the maintenance of Treg cells or induce cell death of activated CD8^+^ T cells. Moreover, IL-15 is the only cytokine recognized to cause antiapoptotic signalling to effector CD8^+^ T cells [85, 86].

IL-15 is a cytokine with proinflammatory activity that seems to provide a protective activity against solid cancers, although its effect could be diverse in the case of MM.

In fact, although IL-15 displays antitumour activities against solid cancers in experimental animal models and has been recognized as one of the most encouraging immunotherapeutic drugs [87–89], only a small number of studies have demonstrated the effectiveness of IL-15 against haematologic tumours.

In a study conducted in MM patients, IL-15 was elevated in the patients compared with the controls. Serum IL-15 levels were increased in MM stage III subjects in comparison with stages I and II, although this difference did not achieve statistical significance. There was a positive correlation between the serum IL-15 and IL-10 concentrations [90].

Investigation of expression models of the IL-15R subunits in six MM cell lines and in the neoplastic cell fraction of 14 MM subjects by Tinhofer et al. showed that malignant plasma cells presented all three elements of the IL-15R heterotrimer [48]. However, whereas healthy B cells from normal subjects downregulate IL-15R*α* after IL-15 administration, MM cells do not show such a decrease in response to IL-15 stimulation. In a different study, IL-15 overexpression in MM plasma cells protected them against apoptosis [48]. These results indicate that MM cells can reduce apoptosis and support themselves via autocrine IL-15 stimulation, thus becoming less dependent upon their microenvironment.

In any case, data from other studies have complicated interpretation of the results.

ALT-803, a fusion protein made by an IL-15 superagonist mutant and a dimeric IL-15 receptor, was discovered to display significantly stronger *in vivo* activity than IL-15 towards T and NK cells. In another study, Xu et al. found that a dose of ALT-803, but not IL-15 alone, eradicated 5T33P and MOPC-315P MM cells in the BM of tumour-bearing mice. ALT-803 therapy significantly augmented the survival of MM-bearing mice and provoked resistance to rechallenge with the same cells via a CD8^+^ T cell-dependent mechanism. ALT-803 therapy stimulated CD8^+^ T cell production of large quantities of IFN-*γ* and augmented the proliferation of CD8^+^CD44^high^ memory T cells *in vivo*.

ALT-803-activated CD8^+^ memory T cells also displayed nonspecific cytotoxicity against MM cells *in vitro*, whereas IFN-*γ* had no direct effects on MM cell growth. The anti-MM activity of ALT-803 was lost in tumour-bearing IFN-*γ* knockout mice [91].

### 4.6. IL-16

IL-16 is recognized to cause chemotaxis of CD4 T cells, eosinophils, and monocytes [92].

Numerous works were able to demonstrate the elevated levels of IL-16 in the BM of MM patients [93, 94]. Nevertheless, the cell types responsible for IL-16 secretion remain undetermined. Alexandrakis et al. indicated that IL-16 is produced by MM cell lines and that augmented IL-16 concentrations were present in the BM of MM patients and post-alloSCT subjects. Furthermore, they also confirmed the presence of a distinct concentration gradient of IL-16 from the PB to the BM. Moreover, IL-16 concentrations were significantly correlated with the grade of BM infiltration by MM cells. Consequently, IL-16 might have a significant role in the pathogenesis of MM [95].

Serum IL-16 was also evaluated before and after the treatment of MM subjects. The concentrations of serum IL-16 in the MM group were much higher than those in the controls. The concentrations of serum IL-16 in the MM subjects who received treatment were all lower than those in MM subjects before therapy, and a correlation as found between concentrations of IL-16 and *β*2-MG [96].

MM cell lines constitutively presented IL-16 and its receptors CD4 and/or CD9 and produced soluble IL-16. Silencing of IL-16 decreased the proliferative ability of MM cells by approximately 80% compared with untreated cells, and the use of a recombinant carboxyl-terminal IL-16 peptide reversed this activity. A monoclonal antibody directed towards IL-16 or its receptor displayed potent proliferation-inhibiting effects on the tumour cells [97].

### 4.7. IL-17

Activated Th17 cells secrete most of the IL-17, although NK cells, CD8^+^ T cells, and neutrophils also generate variable quantities of IL-17. IL-17 stimulates the expression of several chemokines and cytokines, including IL-6, TGF-*β*, matrix metalloproteinase, G-CSF or GM-CSF, and intercellular adhesion molecule-1 in numerous cell types, such as bone marrow stromal cells. It also acts as an inflammation mediator. In fact, this cytokine has a relevant role in the pathogenesis of autoimmune diseases and allergies [98].

Concentrations of IL-17 in MM subjects are higher than those in controls. The levels of IL-17 in ISS I and ISS II stage subjects are not significantly different; the concentration of IL-17 in treated, retreated/refractory subjects are significantly higher than those in subjects with effective treatment, while the concentration in MM subjects is positively correlated with the level of *β*2-MG. Therefore, the IL-17 level may be utilized to determine the ISS stage and therapeutic effectiveness of MM [99].

Finally, miRNAs have been implicated in the pathogenesis of MM [100]. Li et al. evaluated the role of miR-15a/16 in the pathogenesis of MM. They discovered that miR-15a/16 was downregulated in BM-derived mononuclear cells of newly diagnosed MM subjects. Moreover, they demonstrated that miR-15a/16 could reduce IL-17 concentrations in the supernatant of myeloma cells [101].

### 4.8. IL-18

IL-18, an 18 kDa cytokine belonging to the IL-1 family of cytokines, contributes to angiogenesis, immune modulation, and bone metabolism. IL-18 is a powerful proinflammatory cytokine that is capable of stimulating killing by lymphocytes and is crucial to defence against critical infections [102].

IL-18 can provoke both Th1 and Th2 reactions depending on the general cytokine milieu. In fact, with IL-12, IL-18 provokes IFN production, whereas without IL-12, IL-18 causes IL-13 and IL-4 secretion [103].

IL-18 is believed to have angiogenic properties because it can cause endothelial cell migration *in vitro* and blood cell formation.

IL-18 was elevated in MM subjects than in controls [104]. Furthermore, augmented serum IL-18 in MM subjects has been shown to be associated with worse survival, advanced disease, and augmented concentrations of angiogenic cytokines [105, 106]. Significant relationships between IL-18 with VEGF, angiogenin, and bone marrow infiltration have been demonstrated in MM subjects [107].

### 4.9. IL-22

IL-22 is a member of the IL-10 cytokine superfamily, which comprises powerful mediators of inflammatory responses. IL-22 is generally secreted by activated Th1-type T cells, endothelial cells, NK cells, activated dendritic cells, and histiocytes [108, 109]. The transduction of IL-22 signalling is realized by binding to a heterodimeric receptor complex (IL-22R) consisting of IL-22R1 and 2, with successive activation of intracellular kinases (MAP, JAK1, and Tyk2 kinases) and transcription factors, particularly STAT3 [110, 111]. IL-22 has been shown to control the acute-phase response and to stimulate the innate immune system, cell differentiation, cell migration, and gene expression [112–114]. IL-22 may also be secreted, together with IL-17, from splenic tissue inducer-like cells and T_H_ 17 cells, in the presence of several proinflammatory cytokines such as IL-1beta, IL-6, IL-21, and IL-23 [115, 116].

Recently, a novel subset of CD4 T cells has been recognized which produces IL-22 independently of IL-17 [117, 118].

Regarding the role of IL-22 in tumour immunity, IL-22-producing CD4 T cells were discovered in malignant pleural effusion, gastric cancer, pancreatic cancer, colorectal cancer, and B-chronic lymphocytic leukaemia. In gastric cancer, IL-22 levels correlated worse prognosis [119–123].

Di Lullo et al. discovered that the incidence of IL-22 T cells was significantly augmented in PB and BM of stage III and relapsed MM subjects, compared with donors or subjects with asymptomatic or stage I/II MM. Th22 cells derived from the BM of MM subjects produced IL-22 and IL-13 but not IL-17. Moreover, a fraction of the MM cell lines and tumours expressed IL-22RA1 and IL-22-induced STAT3 phosphorylation, cell proliferation, and resistance to drug-induced cell death in MM cells. These data indicate that the augmented frequency of IL-22 T cells is related to a poor prognosis in MM through IL-22 protumour activity, and they suggest that interference with IL-22 signalling pathways could be useful for the treatment of MM [124].

IL-22 was higher in active MM subjects compared with both healthy controls and subjects in remission, as well as in patients who were in remission compared with controls. Moreover, IL-22 levels increased with the disease stage and correlated with IL1-*β*, B22M, and the degree of infiltration. Tsirakis et al. proposed that the augmented concentrations of IL-22 in active MM subjects, in parallel with the disease stage and positively correlating with IL-1beta, might characterize the inflammatory component of the disease. This augmented presence of IL-22 may increase MM growth and, moreover, contribute to the mechanisms responsible for immune deregulation [125].

### 4.10. IL-23

IL-23 is a proinflammatory cytokine that consists of two subunits, p19 and p40. The p40 component is shared with IL-12. However, IL-23 and IL-12 have diverse receptors and actions. While IL-12 stimulates the development of Th1 cells, which secrete IFN, IL-23 is implicated in the differentiation of Th17 cells under proinflammatory conditions, particularly in the presence of transforming growth factor-*β* (TGF-*β*) and IL-6 [126].

The IL-23 receptor consists of the IL-12 receptor b1 chain and the unique IL-23 receptor chain, which is associated with STAT3 and Jak2 [127]. In leukaemic cells and T lymphocytes, IL-23 stimulates activation of STAT family members [127].

IL-23 is generated essentially by myeloid dendritic cells stimulated by Toll-like receptor 2, 4, and 8 ligands and by type 1 macrophages [128, 129]. In fact, IL-23 is considered the principal switch in numerous T cell-mediated inflammatory diseases, while its antitumour effects remain debatable. This proinflammatory cytokine has been shown to impair immune surveillance and augment de novo carcinogenesis and tumour neovascularization [130–132]. However, other researchers have demonstrated that IL-23 exerts antitumour activity by stimulating T and NK cells [133–137].

Regarding MM, although the entire IL-23 receptor is presented on MM cells, it remains unknown whether IL-23 is effective in terms of the modulation of MM cell growth and angiogenesis, stimulation of apoptosis and chemotaxis.

Nevertheless, IL-23 was found to be augmented in MM patients compared with healthy controls [138]. Moreover, IL-23 was associated with decreased CD8 T cell infiltration in the BM microenvironment. These data suggest a possible role of IL-23 in Th17-mediated stimulation of MM cell proliferation and inhibition of immune function [32].

Further complication the situation could be the activity of IL-23 in the genesis of bone diseases in subjects with MM.

Quinn et al. showed that IL-23 decreased osteoclastogenesis indirectly via CD4 T cells and that IL-23p19 decreased bone mass [139]. Kamiya et al. [140] demonstrated that IL-23 was ineffective on RANKL expression and that osteoclastogenesis caused by soluble RANKL was, in part, suppressed by IL-23, whereas the growth of osteoclast progenitors was not altered [140].

These data suggest that under physiologic situations, IL-23 favours high bone mass by reducing bone resorption, while under pathologic conditions, IL-23 has a stimulatory effect on osteoclast formation, mainly via the induction of RANKL by T cells and IL-17 production. In fact, the actions of Th17 and IL-17 in osteoclast activation in MM is well known [141]. As IL-23 is a cytokine implicated in Th17 differentiation and growth, it is reasonable that the high BM concentrations of IL-23 observed in MM subjects promote osteoclastogenesis via the expansion of Th17.

### 4.11. IL-27

IL-27 is a cytokine that belongs to the IL-12 superfamily, which comprises IL-12, IL-23, and IL-35. These cytokines are principally generated by antigen-presenting cells and are implicated in the control of immune responses against infections and cancer development [142].

IL-27 consists of the EBI3 and p35 components and stimulates both STAT1 and STAT3 through different IL-27 receptors, the receptor subunit WSX-1 paired with the gp130 chain [143]. In T lymphocytes, IL-27 stimulates STAT1 and STAT3, thus causing an increase in CD4 T cell growth, stimulation of early Th1 cell differentiation, and suppression of the differentiation of Th17 and of Th2 cells. Moreover, IL-27 plays a role in producing IL-10-regulatory T cells [144].

Plasma cells constitutively present WSX-1 and gp130, and IL-27 stimulates STAT1 phosphorylation while not influencing STAT3 or STAT5 activation [145]. Modifications of STAT activation by IL-27 may reflect the diverse capability of B cell subsets to react to IL-27. For example, IL-27 stimulates the expression of CD86, CD95, and CD54 in activated memory B cells and exhibits chemotactic activity towards plasma cells [145, 146].

Other researchers have confirmed that IL-27 exhibits anticancer activity towards different tumours through several mechanisms, such as through the stimulation of NK cells and the CTL response, as well as the suppression of angiogenesis. This is principally due to the generation of CXCL10 and CXCL9 [71, 76, 147].

Recent data have confirmed the antitumour effects of IL-27 on diverse haematologic malignancies in MM.

Although IL-27 does not modify MM cell growth and apoptosis, a potent decrease in the angiogenic activity of MM cells has undoubtedly been documented. It is well known that MM neoangiogenesis may be stimulated by angiogenic cytokines secreted by tumour and microenvironmental cells [148–150].

IL-27 downregulates a broad panel of proangiogenic factors *in vitro*, comprising VEGF angiopoietins, and matrix metalloproteinases, while upregulating the antiangiogenic factors CXCL9 and CXCL10. Hence, preclinical studies utilizing immunodeficient mice injected with MM cells have shown that IL-27 reduces MM cell growth via suppression of angiogenesis, which causes ischaemic necrosis in tumours. In this model, the principal proangiogenic factors and MM growth factors that were reduced by IL-27 were VEGF-D, IL-6, and CCL2, which also functioned as autocrine and/or paracrine growth factors [12, 151].

### 4.12. TNF

TNF was initially described as a factor that can provoke tumour cell necrosis, but it was later recognized as a proinflammatory cytokine. An incongruous TNF signalling is involved in the pathogenesis of several inflammatory diseases.

TNF binds to two diverse receptors, which are differentially present on cells and tissues and cause both dissimilar and overlapping signal transduction pathways. These different pathways lead to several responses, such as cell death, survival, growth, and differentiation [152].

Nevertheless, not only do the mechanisms decrease the propensity to undergo cancer transformation but also, the mechanisms that increase a tendency towards tumour transformation are intensified by TNF-*α* [153]. Whereas some studies have demonstrated that high concentrations of TNF-*α* reduce tumour angiogenesis in neoplastic tissues, other studies have demonstrated that TNF-*α* may operate as an endogenous tumour growth factor [153].

Binding of TNF to its receptor, TNFR1, results in the temporary formation of a primary membrane-bound signalling complex identified as complex 1, which induces the expression of prosurvival genes. Defective complex I activation causes the induction of cell death (apoptosis or necroptosis), which occurs through the internalization of complex I components and activation of secondary cytoplasmic death complexes known as complex II and necrosome.

Most studies have demonstrated a powerful association between TNF-*α* and haematologic and nonhaematologic malignancy [154].

In fact, at a molecular level, TNF engages NEMO- (nuclear factor-*κ*B (NF*κ*B) essential modulator-) IKK2 (I*κ*B kinase subunit 2, also known as IKK*β*) kinase complex, which stimulates the phosphorylation and degradation of inhibitory I*κ*B*α* (inhibitor of NF-*κ*B*α*), releasing the RelA:p50 dimer into the nucleus though the canonical NF*κ*B pathway [155]. In a negative feedback loop, RelA:p50 transcriptionally stimulates the synthesis of I*κ*B*α*, which guarantees the postinduction decrease in the activity of RelA:p50/NF*κ*B. TNF promotes the transcription of prosurvival factors from their cognate *κ*B-driven promoters. It is usually assumed that RelA:p50 mediates this prosurvival NF*κ*B action in MM cells. Notably, IKK inhibitors have been shown to sensitize MM cells to apoptotic death [156, 157].

Furthermore, Roy et al. reported that MM-associated noncanonical aberrations strengthen prosurvival TNF signalling to cause a prolonged TRAIL-refractory condition. These mutations did not function via a typical p52 NF*κ*B complex but degraded p100 to reposition RelB under I*κ*B*α* control, the degradation of which induced an early RelB:p50-containing NF*κ*B activity [158].

In MM, TNF-*α* is implicated in the production of malignant plasma cells because the plasma cells proliferated when mononuclear cells from MM subjects were exposed to TNF-*α in vitro* [159].

Gene polymorphisms of TNF could also be important for its activity. A study conducted in 94 MM subjects and 141 controls revealed that the A allele of TNF-*α* (-308) was expressed at lower levels in MM subjects. This result indicates that the A allele may have a protective effect against disease [160]. However, another study showed no relationship between MM and this gene polymorphism [161].

However, Basmacı et al. demonstrated that the TNF alpha gene polymorphism (-308) GG genotype was more common in the MM group compared with healthy controls [162].

In a recent study, the GG genotype of TNF-*α* (−238) was shown to be correlated to early progression in MM subjects who had been previously treated with thalidomide- (Thal-) based protocols [163].

Finally, a modification of the concentrations of TNF caused by drugs may play a role in the mechanism of action of the treatments.

In fact, in MM cells, TNF stimulates the expression of prosurvival elements that are known to cause resistance to apoptotic insults [164–166]. Serum concentration of TNF was related to the disease severity in MM [167, 168] and could be a predictive indicator of high symptom burden for subjects undergoing maintenance treatment [169]. Clinical studies have also revealed that Thal analogues, which suppress TNF, are delivered to the tumour microenvironment, augmenting the overall response to TRAIL-based treatment [170, 171]. These data implicate TNF in drug resistance in MM.

Together with the direct effect of lenalidomide (Len) on myeloma growth, both the anti-inflammatory and antiangiogenic effects of Len in the BM environment have been shown to considerably influence the antimyeloma effects of the drug. LEN has an augmented ability to inhibit TNF-*α* delivered by peripheral blood cells compared with Thal [172].

Nevertheless, Len augmented TNF-*α* and IL-8 inflammatory cytokines in MM cells that were both sensitive and resistant to Len [173]. These data suggest that Len treatment induces diverse variations depending on the cell type (MM cells or BMSCs). The effects of Len on TNF-*α* are paradoxical because Len suppresses TNF-*α* production in the BM environment while inducing it in MM cells. The stimulation of TNF-*α* secretion by Len in MM cells occurs irrespectively of the proliferative response to Len.

Analogously, monoclonal antibodies (mAbs) targeting several MM cell surface antigens are under clinical investigation [174]. These mAbs exert antimyeloma action through numerous mechanisms, including an effect on TNF. Elotuzumab is an IgG1 anti-SLAMF7 mAb that is under investigation for therapy in MM [175]. The administration of elotuzumab plus lenalidomide augments myeloma cell killing by modifying NK cell function via the upregulation of TNF-*α*. In coculture assays, TNF-*α* augmented NK cell activation and MM cell death with elotuzumab, and the neutralization of TNF-*α* reduced NK cell activation and MM cell death [176].

#### 4.12.1. TNF Receptors and TNF Family Members

The efficacy of checkpoint inhibitors has confirmed immunomodulatory agents as an important class of antitumour drugs. An interesting costimulatory immunologic target is CD137, or 4-IBB, a component of the TNF receptor superfamily. Binding of 4-1BB provokes an activating signal in CD8 T and NK cells, causing augmented proinflammatory cytokine production, cytolytic activity, and antibody-dependent cell-mediated cytotoxicity [177–179].

Targeting 4-1BB with agonistic monoclonal antibody treatment revealed powerful anticancer actions in tumour models.

An anti-41BB mAb, urelumab, a humanized IgG4 mAb, has been used in the clinic. Urelumab is now being investigated in multiple combinatorial protocols, such as those with elotuzumab in MM [180].

#### 4.12.2. B Cell-Activating Factor (BAFF)

BAFF is a TNF family component that is principally expressed by some T cells, monocytes, and dendritic cells. It is relevant for the preservation of normal B cell development and is considered a survival factor for activated and immature B cells. It is generated as both a soluble protein and a membrane-bound protein. MM cells express BAFF and its receptors [181].

BAFF has been suggested to promote the growth of MM via an autocrine loop [181].

According to the B cell maturation stage, BAFF has been confirmed to stimulate the antiapoptotic proteins Bcl-2 and to decrease the proapoptotic protein Bak.

BAFF has been found increased in MM and correlated with both markers of proliferation and angiogenesis [182–184].

Nevertheless, higher concentrations of BAFF (>1.38 ng/ml) were discovered to be significantly associated with longer OS among MM subjects, which contradicts the data obtained by other authors who proposed BAFF as a potential prognostic factor and a powerful predictor for OS in MM patients due to its correlation with decreased survival [185, 186]. These contradictory data may be due to the diverse ethnic population evaluated, or to diverse types of treatments. Because BAFF controls and increases adaptive and innate immunity, it may cause improved survival in MM subjects [187]. BAFF has also been recognized as one of the principal survival factors for normal plasma cells such as MM cells.

### 4.13. IFN

IFN is produced by several cell populations in the innate and adaptive immune system. Secretion is regulated by antigen-presenting cell- (APC-) secreted cytokines, principally IL-18 and IL-12. IL-4, IL-10, and TGF-*β* negatively control the secretion of IFN. IFN has a relevant action in defence against intracellular pathogens and in immune-mediated inflammatory responses. It causes cytotoxic activity, controls MHC protein expression and antigen presentation, suppresses cell proliferation and apoptosis, and regulates extension of the immune response by stimulating the activation-induced cell death of CD4 T cells [188].

The BM environment in MM subjects has been evidenced high levels of cytokines induced by IFN [3]. Furthermore, numerous action effects of these cytokines could be observed.

For example, IP-10 is a chemokine that is produced by several cells in response to IFN. The receptor of IP-10, CXCR3, is present on normal plasma cells, plasmablasts, and MM cells that control plasma cell migration into the BM [189–191], and it regulates the growth and survival of MM cells [192]. IP-10 is produced by MM cell lines and is augmented in the BM environment of MM subjects compared with controls. Remarkably, BM levels of IP-10 correlated with the stage of MM.

Even the efficacy of some therapies used in MM patients could be mediated by IFN.

The direct anti-MM action of Len has been shown to occur via the induction of G1 growth arrest of MM cells [193] and has consistently been associated with a reduction in IFN regulatory factor 4 [194].

## 5. Anti-Inflammatory Cytokines

### 5.1. IL1-Receptor Antagonist (IL-1Ra)

IL-lRa is produced and released in response to identical stimuli that cause IL-1 release. IL-lRa neutralizes the activity of IL-1 [195].

*In vitro*, IL-1Ra has a better effect than dexamethasone on the reduction of IL-6 secretion; maximal IL-6 suppression and apoptosis induction are attained by the addition of both IL-1Ra and dexamethasone. In a clinical study with IL-1 inhibitors, 3 subjects had a minor response to IL-1Ra alone, 5 subjects attained a partial response, and 4 subjects had a minor response after the addition of dexamethasone. The median overall PFS was 37.5 months. Disease stability was achieved in 8 subjects who received treatment for more than 4 years.

In subjects with latent MM who were at risk of evolution to active myeloma, therapy with IL-1 inhibitors reduced the myeloma growth and CRP concentrations in those who responded, with a chronic disease state and an improved PFS [37].

IL-1 receptor-associated kinase 4 (IRAK4) is a serine/threonine kinase. It has relevant scaffolding and phosphorylation effects on Toll-like receptor (TLR) and IL-1 receptor (IL-1R) signalling pathways. TLR and IL-1R are two cytokine receptors, respectively, which are implicated in inflammatory and immune signalling. Dysregulation of TLR signalling due to IRAK family components has been considered a crucial factor in the initiation of tumours [196].

A combinatorial tactic, targeting IRAK4 and IRAK1, would represent an interesting attempt to decrease cancer progression. Evidence of conjugates that encompass a dual IRAK1/4 kinase inhibitor bound to a Bruton's tyrosine kinase inhibitor resulted in inhibition of NF-*κ*B. In fact, increasing evidence suggests that IRAK inhibitors could be a possible therapy for NF-*κ*B-dependent B cell lymphoproliferative disease Waldenstrom's macroglobulinemia [197, 198].

### 5.2. IL-4

IL-4 is a cytokine that is produced by basophils, Th2 cells, mast cells, and eosinophils. It is the principal stimulus responsible for the increase in Th2-cells and suppression of Th1 development. It also provokes IgE class switching in B cells, augments the expression of class II MHC molecules in B cells and upregulates B cell receptors. IL-4 has a relevant action in the regulation of allergic conditions, as well as the protective response against extracellular parasites [35].

IL-4 serum concentrations are significantly increased in MM subjects, whereas in the BM of MM subjects post-alloSCT, Cao et al. found selectively increased levels of IL-4 [3].

### 5.3. IL-10

IL-10 is likely the most powerful anti-inflammatory cytokine. It is secreted by monocytes/macrophages, NK cells, T and B lymphocytes, and mast cells. As an immunosuppressive cytokine, IL-10 suppresses immune responses by acting on both the innate and adaptive immune system. Therefore, IL-10 can inhibit the secretion of proinflammatory cytokines, antigen presentation, and cell growth [199].

The activities of IL-10 are mediated via the effects of the IL-10 receptor (IL-10R), which includes 2 IL-10R*α* chains and 2 IL-10R*β* chains, on the membrane of the target cell. First, IL-10 reacts with IL-10R*α* due to the superior affinity of IL-10R*α* compared with IL-10R*β*. This contact successively causes an intermediate complex with a binding site for the IL-10R*β* chain. Successive binding of the IL-10R*β* concludes the active receptor complex [200]. This ligand-receptor connection stimulates Janus kinase-1 and tyro-sine kinase-2, which activate tyrosine phosphorylation and STAT3 [201].

IL-10 has a relevant effect on the tumour microenvironment, as it is present on TAMs and CD8+ T cells. IL-10 can be considered an immunosuppressive cytokine, promoting cancer escape from immune surveillance. Moreover, the autocrine path of TAM-derived IL-10 may reduce the expression of the potentially antitumour IL-12 [202]. However, the immunosuppressive effects of IL-10 are not consistent, and they have been proposed to have some immunostimulating faculties, thus playing a relevant role in anticancer response [203–205]. All the above data support the controversial effects of IL-10 in the cancer microenvironment.

IL-10 can considerably increase the growth of B cells, and it has been implicated in their ultimate differentiation into plasma cells, while Il-10 robustly induces immunoglobulin production by plasma cells [206].

IL-10 has been involved in the genesis of malignant B cell diseases such as chronic lymphocytic leukaemia [207]. Moreover, it seems to function as a proliferation factor for MM cells [208], and augmented serum concentrations of IL-10 have been correlated with an advanced MM stage [209].

In one study, the concentration of IL-10 in serum was measured in subjects with newly diagnosed MM. The best cut-off value for IL-10 for predicting survival was 169.69 pg/ml. The overall response rate was 79.2% in patients with low IL-10 levels, which was considerably higher than that in subjects with high IL-10 levels (53.3%). Subjects in the low IL-10 group had superior survival compared with those in the high IL-10 group (3-year PFS rate: 69.3% versus 13.3%; 3-year OS rate: 93.6% versus 51.9%). Multivariate analysis confirmed that high serum IL-10 concentrations at diagnosis was an unfavourable factor for PFS and OS [210].

Alexandrakis et al. evaluated serum concentrations of IL-10 in MM subjects with diverse stages of disease and correlated them with several angiogenic cytokines (VEGF and Ang-2), and with growth parameters such as BAFF and BM infiltration. IL-10 correlated positively with both angiogenic factors and proliferation markers [211].

Finally, both IL-10 and IL-10R single nucleotide polymorphisms (SNPs) were involved in the pathogenesis of many tumours, including haematologic diseases.

Kasamatsu et al. studied the effect of IL-10 −592C/A, IL-10RA I224V, and IL-10RB K47E on the risk of MM and the clinical characteristics of MM. The IL-10RA II genotype was correlated with a haemoglobin concentration lower than that of the IV and VV genotypes. The IL-10 −592 AA genotype was correlated with a superior OS compared to that observed for the CA and CC genotypes. Moreover, differences in survival were observed between subjects treated with thalidomide and/or bortezomib and those cured with conventional drugs. Their results suggest that IL-10 and IL-10R gene polymorphisms may not influence the predisposition to MM but may be correlated with the severity and prognosis of MM [212].

IL-10 increases the proliferation of MM cell lines and MM cells isolated from MM subjects [213]. Gu et al. demonstrated that IL-10 promoted the activation of MM cells by inducing an oncostatin M autocrine loop [214].

Finally, with respect to the pathological action of IL-10 in MM, altered concentrations of IL-10 produced by Treg or MM cells could modulate the host immune response, resulting in a reduction of DC function, by constitutive stimulation of STAT3 in MM [215].

In addition, IL-10 could suppress all-trans retinoic acid- (ATRA-) induced proliferation inhibition of MM cells [216].

### 5.4. IL-11

IL-11 is a glycoprotein-130 (GP-130) cytokine that uses the GP-130 signalling pathway that is shared by several cytokines of the same group. Usually, considered an anti-inflammatory cytokine, IL-11 also functions as a proinflammatory cytokine, supporting its composite role in the immune response. Recently, IL-11 has demonstrated an emergent role in several inflammation-associated tumours. IL-11 is a component of a cytokine group that includes IL-6 and IL-27 [217]. These cytokines are able to activate the Janus kinase (JAK) signal transducer and a STAT3 pathway [218–221].

The binding of IL-11 to its transmembrane coreceptor, IL-11R*α*, has generally been associated with osteoclastogenesis, neurogenesis, adipogenesis, and platelet growth [222]. Nevertheless, recent data indicate the overexpression of IL-11R*α* in prostate cancer, gastric cancer, lung cancer, breast cancer, colorectal cancer, and osteosarcoma, suggesting a relevant effect of IL-11 signalling in the link to inflammation and tumours [223].

Regarding MM, one study showed that IL-11 was present in 26 of 121 MM subjects and in 3 of 28 healthy controls at levels of 1.2 and 0.6 pg/ml [224].

Giuliani et al. has shown that RANK is present in BMSC and endothelial cells but not in MM cells. RANKL did not have a direct effect on MM cell survival, but RANKL treatment caused a relevant augmentation of IL-11 production by both BMSC and endothelial cells. Furthermore, in a coculture model, MM cells upregulated IL-11 production by BMSC and endothelial cells via cell-to-cell contact. However, the presence of the RANK-Fc that blocks the RANK/RANKL interaction suppressed production of IL-11 [225].

The contribution of osteocytes in MM-induced osteoclast (OCL) development and bone lesions remains undetermined. Osteocytes control bone remodelling as a consequence of their cell death-activating OCL recruitment. In another study, the authors discovered that the quantity of viable osteocytes was reduced in MM subjects and negatively related to the number of OCLs. Furthermore, the MM subjects with lytic lesions had significantly fewer viable osteocytes than those without lesions, probably because of augmented apoptosis. A microarray analysis revealed that MM cells modified the transcriptional profiles of preosteocytes by increasing the secretion of osteoclastogenic interleukins such as IL-11 and augmenting their proosteoclastogenic abilities. Finally, the osteocyte presence of IL-11 was higher in MM subjects with than those without lytic lesions [226].

### 5.5. TGF-*β*

TGF-*β* is present as 3 isoforms in mammals: TGF-1, TGF-2, and TGF-3. Platelets are a copious source of TGF [227]. It is produced as a protein complex that requires activation for its biological activity. Once activated, the TGF ligands control cellular processes via the binding of two high-affinity cell-surface receptors, the type I receptor (T RI) and type II receptor (T RII), both of which contain a serine/threonine protein kinase in their intracellular domains [228]. The activated T RI phosphorylates the receptor-activated transcription factors, Smad2/3, which then bind to the common Smad4, translocate into the nucleus, and interact with transcription factors (E2F, Runx1), corepressors (SnoN, c-Ski, SnoN, and TGIF), and coactivators (p300, CBP), to control the transcription of TGF-responsive genes [229, 230].

TGF-*β* is a powerful regulatory cytokine with different effects on haemopoietic cells. This cytokine has a relevant role in inflammation and in inhibition of self-targeted responses [231, 232].

TGF-*β* generally acts to reduce immunoglobulin secretion by B cells [233].

Throughout haematopoiesis, the TGF pathway is a powerful negative regulator of growth-activating differentiation and, when required, apoptosis. In haematologic tumours comprising myeloproliferative disorders, leukaemia, lymphomas, and MM, resistance to these effects of TGF-*β* occurs. Mechanisms underlying this resistance involve interference in the pathway by oncoproteins. These modifications define a tumour suppressor role for TGF in haematologic diseases. However, increased concentrations of TGF can cause myelofibrosis.

In MM, opposition to the homeostatic effects of TGF-*β* signalling arises, perhaps via inadequate trafficking of T*β*RI and T*β*RII to the cell surface. As a consequence, both plasma cells and BM stromal cells from MM subjects produce higher concentrations of TGF-*β* compared with plasma cells from healthy controls [234], participating in the immune alteration present in MM.

Notably, a T*β*RI inhibitor or TGF-*β*-neutralizing antibodies can prevent VEGF and IL-6 production and reduce MM cell proliferation and cell adhesion to BMSCs.

Functionally, the reestablishment of T*β*III expression in MM cells drastically reduced cell proliferation. In a reciprocal manner, shRNA-mediated silencing of endogenous T*β*RIII expression augmented cell proliferation. Although apoptosis was not modified, T*β*RIII reduced growth by stimulating the cyclin-dependent kinase inhibitors p21 and p27. Moreover, T*β*RIII controlled MM cell adhesion, augmenting homotypic MM cell adhesion while reducing MM heterotropic adhesion to BM stromal cells [235].

TGF-*β* is also relevant to hypoxia-induction of MM cancer stem cell-like side populations [236].

Regarding bone disease in MM subjects, TGF-*β* is a powerful inhibitor of terminal OB mineralization [237]. It is secreted by osteocytes and OBs and copiously accumulated in bone matrices in a latent form. It is discharged from bone matrices after bone resorption and activated by matrix metalloproteinases produced by OCs. As osteoclastic bone resorption is augmented in MM, TGF-*β* seems to be plentiful in MM bone lytic lesions, and it may have a relevant role in bone formation altered by MM.

Moreover, TGF-*β*-reduced OB differentiation from BM stromal cells and MC3T3-E1 preosteoblastic cells, as well as reduced adipogenesis from C3H10T1/2 immature mesenchymal cells, supported a differentiation arrest by TGF-*β*. Molecules that were able to inhibit TGF-*β* type I receptor kinase, such as Ki26894 and SB431542, powerfully augmented OB differentiation from BM stromal as well as MC3T3-E1 cells. The reduction of TGF-*β* was capable of reestablishing OB differentiation that had been reduced by MM cell conditioned medium as well as BM plasma from MM subjects. Remarkably, TGF-*β* reduction accelerated OB differentiation in an analogous manner by reducing MM cell proliferation. The effects of anti-MM were due solely to terminally differentiated OBs. Moreover, the reduction of TGF-*β* was capable of reducing MM cell proliferation within the BM while avoiding bone damage in MM-bearing animal models. Research has confirmed that TGF-*β* reduction liberates stromal cells from their differentiation inhibition by MM. TGF-*β* accelerates the formation of terminally differentiated OBs that increase the sensitivity of MM cells to anti-MM drugs to overwhelm the drug resistance due to stromal cells [237].

Although TGF-*β* increases the growth of osteoblast progenitors, it strongly reduces later phases of osteoblast maturation and suppresses matrix mineralization. Reduction of TGF-*β* signalling can become a novel therapeutic method against MM [237].

TGF-*β* could also be implicated in chemoresistance. Frassanito et al. showed that BM cancer-associated fibroblasts (CAFs) from bort-resistant subjects are insensitive to bort and defend RPMI8226 and subject plasma cells against bort-induced apoptosis [238]. Bort stimulates CAFs to secrete high concentrations of TGF-*β*. In the syngeneic 5T33 MM model, bort therapy caused an increase in LC3-II^+^ CAFs. TGF-*β* facilitated bort-induced autophagy, and its block by LY2109761, a selective T*β*RI/II inhibitor, decreased the presence of LC3-II and p-Smad2/3 and induced apoptosis in bort-resistant CAFs. Bort and LY2109761 synergistically provoked apoptosis of RPMI8226 cocultured with bort-resistant CAFs [239].

Progress in the TGF signalling field should reveal new possibilities for the treatment of MM [239].

## 6. Mediators of Cytokines

### 6.1. Heat-Shock Proteins

Heat-shock proteins (HSPs) are believed to be highly conserved proteins and a danger signal that chaperone, fold, and transport proteins when cells are subjected to numerous stresses. Augmented production of extracellular HSPs causes the liberation of proinflammatory cytokines by macrophages and monocytes. This provokes upregulated expression of antigen-presenting molecules on immature DCs and changes the ability of these cells to participate in the immune response [240]. Furthermore, HSPs represent the endogenous signals that stimulate DCs as they translocate antigen to the cytosol in DCs [241]. These actions can be either protective, such as after a cellular insult, or damaging because they can lead to disproportionate inflammation [242].

Under nonstressed situations, chaperones are implicated in numerous crucial biochemical activities. They support the exact folding of the polypeptide as translation progresses, control the transport of proteins across subcellular membranes, influence the turnover of folded proteins, and contribute to the posttranslational control of signalling proteins, avoiding their irregular aggregation and helping client proteins avoid destruction via the ubiquitin-proteasome pathway.

Despite their name, most of these substances are ubiquitously present under physiological situations. However, their synthesis is augmented by a large range of stressful situations beyond heat shock, and their presence has been found to be significantly augmented in numerous tumours (both solid cancers and haematologic diseases) [243, 244].

The HSP90 protein family comprises HSP90a (HSPC1), HSP90b (HSPC3), and gp96 (HSP4).

The heat-shock protein 90 kDa appears to be one of the most interesting because it interacts with several client proteins that are implicated in numerous relevant regulatory pathways, such as cell cycle control and defence against apoptosis [245, 246]. Furthermore, its action appears to be essential for cancer cells to preserve an abnormal homeostasis, defending themselves against the microenvironment, which is acidotic, hypoxic, and nutrient-deprived [247, 248].

Tumour cell apoptosis is controlled by HSP90, principally through its action on TNF-mediated signalling pathways [249] and on nuclear factor-*κ*B [250]. It has also been observed that some HSP90 clients, such as p53 and SRC tyrosine kinase, often assume oncogenic mutations that lead to an abnormal interaction with chaperones [251]. This molecular connection appears to inhibit the process of p53-ubiquitylation and enzymatic degradation, altering cell cycle control [252, 253].

HSP90 is overexpressed in MM and promotes tumour cell survival. Augmented HSP90 protein concentrations were demonstrated in IL-6 transgenic mice that display increased IL-6 concentrations. Similarly, it has been shown that IL-6 can provoke augmented concentrations of HSP90 in numerous cell types. Moreover, it has been established that STAT3 and CCAAT/enhancer-binding protein *β* (C⁄EBP*β*) bind to and activate the HSP90*β* promoter and augment HSP90 levels [254, 255].

Pharmacologic blockade of HSP90 has been found to provoke MM cell death [256, 257].

Numerous studies have demonstrated the effectiveness of HDAC inhibitors in curing MM [258, 259]. Vorinostat increased p21^WAF1^ by changing the methylation and acetylation of core histones and by impeding the enzyme accessibility of DNase I in the promoter region of MM cells [260].

Panobinostat, a pan-HDAC inhibitor, with Bor and dexamethasone, has attained long progression-free survival in MM subjects. Panobinostat reduced MM cell proliferation by destroying protein phosphatase 3 catalytic subunit a (PPP3CA), a catalytic subunit of calcineurin. This modification was proposed to be mediated by blocking the function of heat-shock protein 90 due to HDAC6 inhibition [261].

Xie et al. produced an MM cell line, J558HSP, presenting endogenous P1A tumour antigen and a transgenic form of membrane-bound HSP70 and heat-shocked J558HS expressing cytoplasmic HSP70, and purified EXOHSP and EXOHS from the J558HSP and J558HS tumour cell culture supernatant. They confirmed that EXOHSP was able to cause maturation of DCs and to stimulate Th1 cell responses [262].

Jung et al. examined whether treatment of MM cells with a STAT3 inhibitor (JSI-124) and/or Bor before loading into DCs could influence DC function. The therapy with JSI-124 and Bor caused the highest expression of HSP 90 and the lowest expression of p-STAT3 in dying MM cells. DCs loaded with JSI-124 and Bor produced MM-specific cytotoxic T lymphocytes (CTLs) [263].

### 6.2. Leptin and Resistin

Accumulating evidence supports a role for obesity in the genesis of MM [264]. As adipose tissue increases in obesity, the quantities of anti-inflammatory adipokines are reduced and the quantities of proinflammatory adipokines with oncogenic capability, such as resistin, leptin, visfatin, and chemerin, are augmented [265].

Leptin is a crucial regulator of energy expenditure and caloric intake, and numerous studies have correlated obesity to altered leptin metabolism [266].

Moreover, a correlation between leptin and the immune system has been discovered, and a correlation between plasma leptin concentrations and the TNF-*α* system has been observed in obese patients [267, 268].

Hofmann et al. found that MM subjects had higher concentration of leptin in comparison to controls, although this difference did not achieve statistical significance. They subsequently concluded that leptin concentrations were not associated with MM risk [269].

However, in another study, leptin was elevated in MM subjects compared with the healthy controls. A significant positive correlation was discovered between IgG levels and leptin. Moreover, a significant difference in leptin concentration has been observed between stage I and stage II [270].

Finally, Alexandrakis et al. confirmed an increase of leptin levels in newly diagnosed MM patients, and they found a decrease in leptin following treatment [271].

Resistin was initially identified as a molecule that provoked insulin resistance and produced hyperglycaemia without influencing peripheral insulin sensitivity [272].

Regarding resistin and MM, Considine et al. discovered that the concentration of resistin was lower in MM subjects with respect to the control group, but this difference did not attain significance. Moreover, they found insignificant correlations between resistin and IgG concentrations and between BM plasma cells and resistin in MM patients. Only LDH levels had a negative correlation with the resistin level [273].

## 7. Discussion

### 7.1. A New Therapeutic Target: Cytokines

The role of cytokines in the pathogenesis and progression of neoplastic diseases is now undeniable. Consequently, we could employ cytokines as therapeutic targets with numerous benefits. First, proteins that regulate the inflammatory process can be suppressed. Moreover, cytokines are well validated in animal models utilizing genetic models such as knockout mice or neutralizing antibodies.

Nevertheless, the disadvantages of cytokine treatment derive from the same properties. Cytokines influence numerous processes in parallel. Moreover, they have redundancy, and the effects attained by suppressing one specific cytokine can be balanced by others. Alterations of the cytokine system may lead to a modulated immune response. For example, the suppression of proinflammatory cytokines can cause compromised host defence against infections, while the suppression of regulatory cytokines can provoke autoimmunity or tissue damage. Moreover, the fabrication of biologics is still a high-priced process since their manufacturing requires sterile conditions and multiple phases of purification, and recombinant cytokines have a restricted half-life, necessitating special storage conditions [274].

However, numerous drugs utilized in MM therapy have an effect on cytokines. Len exerts cytotoxic actions on MM cells and has anti-inflammatory, immunomodulatory, and antiangiogenic actions on BM accessory cells. Its immunomodulatory actions comprise the stimulation of subsets of T cells to secrete Th1 cytokines such as IL-2 and IFN-*γ* while suppressing the production of Th2 cytokines such as IL-6 and TNF-*α* [275–277].

Of interest could be the data showing transient inflammatory reactions in a subpopulation of MM subjects during Len plus dexamethasone treatment. Adjustment of Th1 and Th2 cytokine secretion by Len may participate in the transitory inflammatory reaction in MM patients [278].

Finally, considering inflammation and cytokines as possible targets, it is possible to consider the possibility of introducing new drugs in MM therapy.

Cyclooxygenase 2 (COX-2) is an inflammation-associated enzyme. Generally, Cox-2 is not present in cells, but its expression can be increased in an environment including growth factors, cytokines, and inflammatory molecules [279].

There are few studies reporting Cox-2 expression in MM subjects [280–282]. Moreover, other papers showing Cox-2 expression in MM cell lines are contradictory [283, 284].

It has been suggested that chronic inflammation is linked to aberrant angiogenesis [19]. Khan et al. demonstrated a positive correlation between angiogenic factors and cyclooxygenase [285].

Targeting COX-2 by utilizing inhibitors that establish antiangiogenic and antitumour effects could be used as a novel treatment approach for MM therapy.

### 7.2. Multiple Myeloma and Inflammation: A Nonunique Connection

However, the relationship between inflammation and cancer and between cytokines and neoplasms is certainly less linear and defined than previously thought, and it is very different than that observed for other pathophysiological conditions such as ageing [286, 287].

The immune system can defend against tumours, and several cytokines predict long-term survival for subjects with advanced cancer.

Proinflammatory cytokines such as IL-6 and IL-1 are believed to be indispensable for cancer progression, and anti-inflammatory drugs have been proposed to treat tumours. Nevertheless, anti-inflammatory therapies may theoretically reduce protective antitumour immunity. In fact, although inflammation is commonly deemed to be cancer promoting, few studies in breast, bladder, and colorectal cancer suggest that cancer infiltration by inflammatory cells may be correlated with a better prognosis [288–290].

As previously shown, proinflammatory cytokines can have both pro and anticancer activities, while cytokines with potent anti-inflammatory activity may strongly favour the growth of tumours. To bring together these contrasting views, it is possible to suggest that inflammation, when guided by cancer-specific Th1 cells, may inhibit tumour onset and progression. In a Th1 microenvironment, proinflammatory cytokines (e.g., IL-6, IL-1*α*, and IL-1*β*) may contribute to tumour eradication by attracting leucocytes from the circulation and by increasing CD4 ^+^ T cell activity.

Approaches to fight cancer should be based on promoting rather than reducing the immune response against tumours. Thus, it is essential to better comprehend the relationship between immune cells, inflammation, and cancer.

MM is typically exemplified by a desynchronized cytokine system with an increase in inflammatory cytokines.

Ben-Sasson et al. evaluated locally produced cytokines throughout the primary immune response against MM in mice [291]. Strikingly, efficacious tumour immunosurveillance due to tumour-specific CD4 ^+^ T cells was consistently related to increased local concentrations of both proinflammatory (IL-6, IL-1*α*, and IL-1*β*) and Th1-associated cytokines (IL-2, IL-12, and IFN-*γ*).

Tumour suppression is attained by the cooperation of cancer-specific Th1 cells and cancer-infiltrating, antigen-presenting macrophages. Th1 cells provoke the production of IL-6 and IL-1*β* by macrophages. Th1-derived IFN-*γ* is known to cause macrophage cytotoxicity to tumour cells and to stimulate macrophages to produce the angiostatic factors CXCL10/IP-10 and CXCL9/MIG. Thus, inflammation, when guided by cancer-specific Th1 cells, may inhibit rather than stimulate tumours.

To confirm this statement, Haabeth et al. utilized a technique to measure locally produced cytokines during primary anticancer immune responses in mice [292]. Employing this approach, they recognized a core of nine cytokines that consistently correlated with efficacious tumour suppression: IL-12p70, IFN-*γ*, IL-1*α*, IL-1*β*, IL-2, IL-3, IL-6, CXCL10, and CXCL9. The finding that IL-12 and IFN-*γ* are consistently associated with tumour rejection is coherent with a Th1 polarization of the immune response, which is generally believed to be advantageous for immunological control of tumours [293, 294]. In contrast, the proinflammatory cytokines IL-6, IL-1*α*, and IL-1*β* may appear more unexpectedly as chronic inflammation related to the tumour [295–298].

The finding that increased concentrations of IL-1 were connected with efficacious tumour immune-surveillance is of special interest. IL-1 is a canonical proinflammatory cytokine, and it acts as a positive feedback loop in inflammation. IL-1 has been demonstrated to increase the growth and differentiation of CD4 ^+^ T cells and to stimulate macrophage tumouricidal action *in vitro* [299]. Significantly, IL-1*β* production by macrophages is reliant on activation of the inflammasome, a cytosolic molecular complex responsible for producing active IL-1*β* by cleaving the inoperative precursor. The inflammasome acts as a sentinel by identifying pathogens and danger signals [300]. In cancer immunosurveillance, the type of endogenous danger signals identified by the inflammasome remain to be clarified, although a role for ATP produced by necrotic tumour cells has been proposed [301].

Hence, caution should be used when considering therapies that target factors with pro or anti-inflammatory activity. Drugs that may reduce the tumour-suppressive Th1-driven inflammatory immune response should be avoided.

New perspectives concerning intervention seem possible, and the use of nanotechnology could be a powerful approach to the use of cytokines in the prevention and treatment of cancer [302–304]. A better understanding of the relationship between inflammation and myeloma will ensure more effective therapeutic interventions.
